# Estrogen receptor β inhibits breast cancer cells migration and invasion through CLDN6-mediated autophagy

**DOI:** 10.1186/s13046-019-1359-9

**Published:** 2019-08-14

**Authors:** Peiye Song, Yanru Li, Yuan Dong, Yingying Liang, Huinan Qu, Da Qi, Yan Lu, Xiangshu Jin, Yantong Guo, Yiyang Jia, Xinqi Wang, Wenhong Xu, Chengshi Quan

**Affiliations:** The Key Laboratory of Pathobiology, Ministry of Education, College of Basic Medical Sciences, Jilin University, 126 Xinmin Street, Changchun, 130021 Jilin China

**Keywords:** Estrogen receptor β, CLDN6, Autophagy, Migration, Invasion, Breast cancer

## Abstract

**Background:**

Estrogen receptor β (ERβ) has been reported to play an anti-cancer role in breast cancer, but the regulatory mechanism by which ERβ exerts this effect is not clear. Claudin-6 (CLDN6), a tight junction protein, acts as a tumor suppressor gene in breast cancer. Our previous studies have found that 17β-estradiol (E2) induces CLDN6 expression and inhibits MCF-7 cell migration and invasion, but the underlying molecular mechanisms are still unclear. In this study, we aimed to investigate the role of ERβ in this process and the regulatory mechanisms involved.

**Methods:**

Polymerase chain reaction (PCR) and western blot were used to characterize the effect of E2 on the expression of CLDN6 in breast cancer cells. Chromatin immunoprecipitation (ChIP) assays were carried out to confirm the interaction between ERβ and CLDN6. Dual luciferase reporter assays were used to detect the regulatory role of ERβ on the promoter activity of CLDN6. Wound healing and Transwell assays were used to examine the migration and invasion of breast cancer cells. Western blot, immunofluorescence and transmission electron microscopy (TEM) were performed to detect autophagy. Xenograft mouse models were used to explore the regulatory effect of the CLDN6-beclin1 axis on breast cancer metastasis. Immunohistochemistry (IHC) was used to detect ERβ/CLDN6/beclin1 expression in breast cancer patient samples.

**Results:**

Here, E2 upregulated the expression of CLDN6, which was mediated by ERβ. ERβ regulated CLDN6 expression at the transcriptional level. ERβ inhibited the migration and invasion of breast cancer cells through CLDN6. Interestingly, this effect was associated with CLDN6-induced autophagy. CLDN6 positively regulated the expression of beclin1, which is a key regulator of autophagy. Beclin1 knockdown reversed CLDN6-induced autophagy and the inhibitory effect of CLDN6 on breast cancer metastasis. Moreover, ERβ and CLDN6 were positively correlated, and the expression of CLDN6 was positively correlated with beclin1 in breast cancer tissues.

**Conclusion:**

Overall, this is the first study to demonstrate that the inhibitory effect of ERβ on the migration and invasion of breast cancer cells was mediated by CLDN6, which induced the beclin1-dependent autophagic cascade.

**Electronic supplementary material:**

The online version of this article (10.1186/s13046-019-1359-9) contains supplementary material, which is available to authorized users.

## Background

Estrogen plays an important role in hormone-dependent breast cancer progression and metastasis. The effects of estrogen are primarily mediated through the estrogen receptors (ERs), ERα and ERβ [1]. The contribution of ERα to the normal development of the mammary gland and the tumorigenesis and progression of breast cancer is essential [2]. ERα expression in normal breast epithelial cells is approximately 10% but increases to 50–80% in breast cancer cells [3]. Loss of ERα in breast cancer patients indicates poor prognosis, and ERα has been the principal biomarker for endocrine therapy in breast cancer [4]. However, only 70% of ERα-positive breast cancers respond to tamoxifen (ER antagonist) treatment, and 30–40% of patients relapse during treatment and become resistant to endocrine therapy [5]. ERβ has the same structural domains as ERα, but its function is not exactly the same as ERα. The role of ERβ in breast cancer remains elusive, and ERβ is currently not used in the diagnosis or treatment of breast cancer patients [6]. Although a few studies claim that ERβ expression promotes the invasion and metastasis of breast cancer and that high ERβ level is linked with poor prognosis [7], multiple studies have demonstrated that ERβ is an anti-oncogene in breast cancer. In contrast to those of ERα, clinical studies showed that the levels of ERβ were high in mammary epithelial tissues and decreased during tumor progression [3]. In triple negative breast cancer (TNBC), high expression of ERβ was significantly associated with good clinical outcome in patients treated with tamoxifen [8]. In vitro studies showed that ERβ expression inhibited the cell proliferation and the migratory and invasive properties of breast cancer cells [9, 10]. Therefore, ERβ may be a potential target for novel therapeutic avenues in breast cancer. Nevertheless, the molecular mechanisms underlying the inhibitory effects of ERβ on breast cancer remain unidentified and need to be explored.

Recent studies have suggested that ERβ could also trigger autophagy [11, 12]. Autophagy plays a key role in the maintenance of cellular homeostasis [13]. Dysregulation of autophagy has been implicated in cancers. A recent study reported that ERβ activation could inhibit breast cancer cell proliferation by reducing the G2/M phase as well as triggering autophagy [11]. ERβ-induced damage regulated autophagy modulator 2 (DRAM2)-mediated autophagy has been associated with a reduction of cancer cell proliferation in Hodgkin lymphoma (HL) cells [12]. However, few studies have reported that the inhibitory role of ERβ on migration and invasion is directly related to the modulation of autophagy in breast cancer. Furthermore, the regulatory mechanism of ERβ induced-autophagy is still unclear. Intriguingly, in this study, we found that ERβ induced autophagy and inhibited migration and invasion through claudin-6 (CLDN6) in breast cancer cells.

CLDN6 is a tight junction (TJ) protein that belongs to a family of transmembrane proteins with 4 transmembrane domains, and 27 members of this family have been identified [14, 15]. As an important component of TJs, CLDNs not only play important roles in the classic barrier and fence functions of TJs but are also involved in regulating cellular communication and signaling [16]. CLDNs possess a carboxy-terminal PDZ-binding motif. This domain allows CLDNs to interact with cytoplasmic scaffolding proteins (PDZ domain-containing proteins), such as zonula occludens (ZO-1) and afadin, which are important for CLDNs to communicate with a multitude of signaling proteins [17]. In previous studies, our group first cloned the CLDN6 gene from mammary epithelial cells of COP rats and identified CLDN6 as a breast cancer suppressor gene [18, 19]. We have reported that CLDN6 expression induces apoptosis and inhibits tumor growth, migration and invasion in breast cancer cells [20–22]. Moreover, in our recent studies, we found that CLDN6 overexpression not only strengthened the tight junctions in breast cancer cells but also induced a large number of autophagic vacuoles observed under transmission electron microscopy (TEM). A series of subsequent experiments demonstrated that CLDN6 induced autophagy, whereas the relationship between CLDN6-induced autophagy and breast cancer remains poorly investigated.

Our previous studies have shown that 17β-estradiol (E2) upregulates CLDN6 expression and hinders MCF-7 cell migration and invasion, but the molecular mechanisms are still unclear. Interestingly, in this study, we found that the expression of CLDN6 was increased and migration and invasion were hindered in both MCF-7 (ERα+/ERβ+/GPR30+) and MDA-MB-231 (ERα−/ERβ+/GPR30-) cells after E2 treatment. Thus, we supposed that this E2-induced effect was not ERα-dependent, and we wanted to explore the role of ERβ in this process. In view of the abovementioned reports, we presumed that ERβ regulated CLDN6 expression and that ERβ-induced autophagy affected migration and invasion in breast cancer cells. In addition, little is known about the role of CLDN6 in ERβ-induced autophagy. Hence, the current study aimed to explore the regulatory role of ERβ on CLDN6 expression in breast cancer cells and the mechanism related to its biological functions. In this study, we demonstrated a novel finding that ERβ induced autophagy and inhibited migration and invasion via CLDN6 in breast cancer cells. We also analyzed the molecular mechanisms underlying this effect in some details.

## Methods

### Cell culture and reagents

The human breast adenocarcinoma cell lines MCF-7, MDA-MB-231 and SK-BR-3 were obtained from the Cell Bank of the Chinese Academy of Sciences (Shanghai, China) and cultured in DMEM (GIBCO, USA) supplemented with 10% fetal bovine serum (FBS) (Biological Industries, Israel) at 37 °C with an atmosphere of 5% CO_2_. For estrogen-free cell culture, cells were cultured in phenol red-free DMEM and serum-free (HyClone, USA) for 12 h before specific treatments. The 17β-estradiol (E2) (10006315) and PHTPP (16025) were purchased from Cayman Chemical (Denver, USA). Diarylpropionitrile (DPN) (H5915) was purchased from Sigma (St. Louis, MO, USA). ICI 182.780 (HY-13636), Chloroquine (CQ) (HY-17589) and 3-methyladenine (3-MA) (HY-19312) were purchased from MCE (New Jersey, USA).

### RT-PCR and quantitative real-time PCR (qRT-PCR)

Total RNA was isolated from cells using TRIzol (Invitrogen, Carlsbad, CA, USA) and converted to cDNA using First-Strand cDNA Synthesis kit (TransGen Biotech, Beijing, China). Semiquantitative RT-PCR and qRT-PCR were carried out as Liu et al. [23] described previously. The primers were synthesized by Sangon (Shanghai, China). PCR primer sequences were as follows:

- CLDN6 (PCR)-forward: TTCATCGGCAACAGCATCGT,
- CLDN6 (PCR)-reverse: GGTTATAGAAGTCCCGGATGA;
- CLDN6 (qRT-PCR)-forward: CCCTTATCTCCTTCGCAGTG,
- CLDN6 (qRT-PCR)-reverse: ATGCTGTTGCCGATGAAAG;
- β-actin-forward: CAGAGCCTCGCCTTTGCCGATCC,
- β-actin-reverse: CCTTGCACATGCCGGAGCCGT .

### Western blot analysis

Protein extraction and western blot analysis were performed as described by Yang et al. [24]. The following primary antibodies were used: CLDN6 (V118, 1:1000) (Bioworld Technology, USA); ERα (ab66102, 1:1000), ERβ (ab288, 1:1000) (Abcam, Cambridge, UK); LC3B (D11, 1:1000), beclin1 (D40C5, 1:1000), Atg5 (D5F5U, 1:1000), Atg16L1 (D6D5, 1:1000) (Cell Signaling Technology, Danvers, MA, USA); ZO-1 (21773–1-AP, 1:1000), UVRAG (19571–1-AP, 1:1000), β-actin (60008–1-Ig, 1:5000) (Proteintech Group, USA). The signals were visualized using ECL reagent (Millipore, Billerica, MA, USA).

### Wound healing assay

The cells from each group were seeded onto 6-well culture plates with corresponding treatment. When cells had grown to full confluence, wounds were created through the monolayers by using a sterile pipette tip. The wounded areas were imaged at 0 h and 24 h by an inverted microscope (Olympus, Japan). Image J software (NIH, USA) was employed to analyze the wound widths from the images.

### Transwell assay

Transwell chambers (Costar, USA) with matrigel were used to perform cell invasion assays. An equal number of cells (1 × 10^5^ cells) were loaded into matrigel (BD Biosciences, USA) precoated chambers in 200 μl serum-free media. NIH/3 T3 conditioned medium as the chemoattractant was placed in the lower compartment of the chamber. After 24 h incubation, cells were fixed in 4% paraformaldehyde and stained with 0.1% crystal violet. No invading cells were removed with cotton swabs. Images were photographed in three randomly selected fields of view at 200 × magnification. NIH/3 T3 conditioned medium was prepared by culturing NIH/3 T3 fibroblasts cells for 48 h in serum-free media [25]. The supernatants were collected and stored at − 80 °C.

### Immunofluorescence

Cells were grown on coverslips and then incubated with or without DPN for 24 h and fixed in 4% formaldehyde. The cells were blocked and incubated with primary antibodies CLDN6 (1:200), ERβ (1:200) and LC3B (1:500) overnight at 4 °C. Cells were stained with Alexa Fluor® 594/488 goat anti-rabbit IgG (Cell Signaling Technology, Danvers, MA, USA) for 1 h at room temperature. Finally, samples were counterstained with 4, 6-diamino-2-phenylindole (DAPI) (Sigma). Images were taken with fluorescence microscopy (Olympus, Japan).

### Transmission electron microscopy (TEM)

Cells were exposed to DPN with or without 100 nM for 24 h. The treated cells were fixed with 4% glutaraldehyde and post-fixed in 1% OsO4. Samples were dehydrated through a graded series of ethanol solutions and embedded in Eponate 12 epoxy resin. Ultrathin sections were counterstained with uranyl acetate and lead citrate. Observation and photography carried out by transmission electron microscopy (FEI Tecnai Spirit, USA).

### Transfection

Three candidate target sequences of ERβ were cloned into U6-MCS-Ubiquitin-Cherry-IRES-puromycin vectors (GeneChem, Shanghai, China). Scrambled RNAs were used as a negative control (NC) for nonsequence-specific effects. Information on CLDN6 overexpression and CLDN6 short hairpin RNA (shRNA) plasmids has been described previously [26]. The ERβ overexpression plasmid was generated by cloning ERβ cDNA into the pLenti-CMV-GFP-Puro vector (PPL00807-4a, PPL Genebio Technology, Nanjing, China). Beclin1 shRNA was cloned into the pPLK/GFP + Puro vector (PPL00043-3b, PPL Genebio Technology, Nanjing, China). All lentiviral plasmids were cotransfected into 293 T cells together with packaging plasmids (pMD2.G and psPAX2). The supernatants containing lentiviral particles were collected 48 h after transfection and used to transduce cells according to the manufacturer’s protocol of Lipofectamine 2000 (Invitrogen, USA). The target for human ERβ shRNA sequences were as follows,

- shRNA-1:5′- TGCTTTGGTTTGGGTGATT − 3′,
- shRNA-2: 5′- TTCTCCTTTAGTGGTCCAT − 3′,
- shRNA-3: 5′- GTAAACAGAGAGACACTGAAA − 3′.

The target for human beclin1 shRNA was 5′- GTGGACACGAGTTTCAAGATT − 3′.

### Nuclear/cytosol fractionation assay

2 × 10^6^ MDA-MB-231 cells were seeded in 10-cm culture plates and treated with DMSO (control) or DPN (100 nM) for 24 h, respectively. The nuclear and cytosolic fractions were isolated with a Nuclear/Cytosol Fractionation Kit (Beyotime Biotechnology, Shanghai, China) according to the manufacturer’s instructions. The subcellular protein extracts were then analyzed by western blot.

### Chromatin immunoprecipitation (ChIP) assay

MDA-MB-231 cells were grown in 10-cm culture plates and cultured with serum-free medium for 12 h, and then treated with 100 nM DPN for 24 h. Following treatment, ChIP assay was performed as described previously [23] with minor modifications. The precleared chromatin was incubated overnight with ERβ (ab288) (Abcam, Cambridge, UK) or Sp1 (D4C3) (Cell Signaling Technology, Danvers, MA, USA) and immunoprecipitated with protein A/G magnetic beads. A normal rabbit IgG (Millipore, Billerica, MA, USA) was used as negative control. Amplified DNA fragments were visualized on a 2% agarose gel. PCR primer sequences were as follows:

- ChIP-CLDN6–1 forward: GCTTAAGTGGTGAAGCGGAG,
- ChIP-CLDN6–1 reverse: CAGACGTCCAGACTCACCCA;
- ChIP-CLDN6–2 forward: TGTGCGTGTTGGAGAGACG,
- ChIP-CLDN6–2 reverse: CGAAGGACCCTATCACCTCG.

### Dual luciferase reporter assay

MDA-MB-231 cells were seeded in 6-well culture plates, and transfected with pGL3-CLDN6 plasmid which containing CLDN6 promoter fragment (− 2000/+ 250 bp) and renilla luciferase reporter plasmid (pRL-TK). After transfection for 48 h, cells were treated with DPN. Luciferase activities were measured using the dual-luciferase reporter assay system (Promega, San Luis Obispo, CA, USA) according to the manufacturer’s protocol. Firefly luciferase activity was normalized to renilla luciferase activity. Reporter plasmids were purchased from GeneChem Co., Ltd. (Shanghai, China).

### Co-immunoprecipitation (co-IP) assay

After DPN treatment, cells were harvested and lysed with NP-40 lysis buffer. Protein lysates were incubated with the anti-UVRAG/anti-ZO-1 antibody or normal rabbit IgG, and rotated overnight at 4 °C to form the immune-complex. Reaction mixture was incubated with protein A/G plus-agarose beads (Santa Cruz Biotechnology, Santa Cruz, CA, USA) for 3 h at 4 °C. Agarose beads were washed five times with cold washing buffer and heated with 40 μl 1 × SDS buffer to 100 °C for 5 min. The samples were analyzed by Western blot.

### Immunohistochemistry (IHC)

Human breast cancer tissue specimens (HBreD070CS02) were purchased from Shanghai Outdo Biotech CO. The tissues (*n* = 70) including 11 paracancerous tissues, 4 intraductal carcinoma and 55 invasive ductal carcinoma. The primary antibodies against ERβ (ab288, 1:200, Abcam), CLDN6 (V118, 1:200, Bioworld Technology) and beclin1 (D40C5, 1:200, CST), respectively, and then incubated at 4 °C overnight in a humidified container. Following washing three times with phosphate buffer saline (PBS), the section was treated with UltraSensitive™ SP (Mouse/Rabbit) IHC Kit according to the manufacturer’s instructions (KIT-9710, MXB Biotechnology, Fuzhou, China). 3, 3′-diaminobenzidine (DAB) was used for color development. Immunostaining was evaluated by two pathologists using a blind protocol design. For ERβ, CLDN6 and beclin1, each tissue sample was scored according to its staining intensity (0, none; 1, weak; 2, moderate; 3, strong) multiplied by the point of the percentage of stained cells (positive cells ≤25% of the cells: 1; 26–50% of the cells: 2; 51–75% of the cells: 3; ≥75% of the cells: 4). The range of this calculation was 0–12. The median value of scores was employed to determine the cut-off. Cancers with scores above the cut-off value were considered to have high expression of the indicated molecule and vice versa.

### Animal studies

An experimental model of lung metastasis was used to investigate the effects of CLDN6-beclin1 axis on breast cancer metastasis. Three groups of five female Balb/c nude mice (4–6 weeks old and 18–20 g) were maintained in an environment with a standardized barrier system of the Experimental Animal Center of Jilin University. One group was injected with the MDA-MB-231/NC cells, the second group was injected with the MDA-MB-231/CLDN6 cells and the third group was injected with the MDA-MB-231/CLDN6-sh-beclin1 cells. For each nude mouse, 1 × 10^6^ cells in 100 μl PBS were injected via the tail vein. After 4 weeks of observation, mice were sacrificed. The lungs and major organs were removed and observed by using a fluorescence imager (IVIS Spectrum, Caliper Life Sciences). Then these organs were fixed, embedded, and sectioned. The metastasis ability was detected by hematoxylin and eosin (H&E) staining.

### Statistical analysis

All statistical analyses were performed using the SPSS 13.0 (SPSS Inc., USA) statistical software and GraphPad Prism 7.0 (GraphPad, USA). The statistical significance was analyzed by one-way ANOVA or Student’s *t*-test. The data were presented as the mean ± standard deviation (SD) at least three independent experiments. Categorical data were analyzed by Fisher’s exact test or chi-square test. The correlations were analyzed using Pearson’s correlation coefficients. *P* < 0.05 was considered statistically significant.

## Results

### E2 upregulates CLDN6 expression and inhibits the migration and invasion of breast cancer cells

**Fig. 1:**
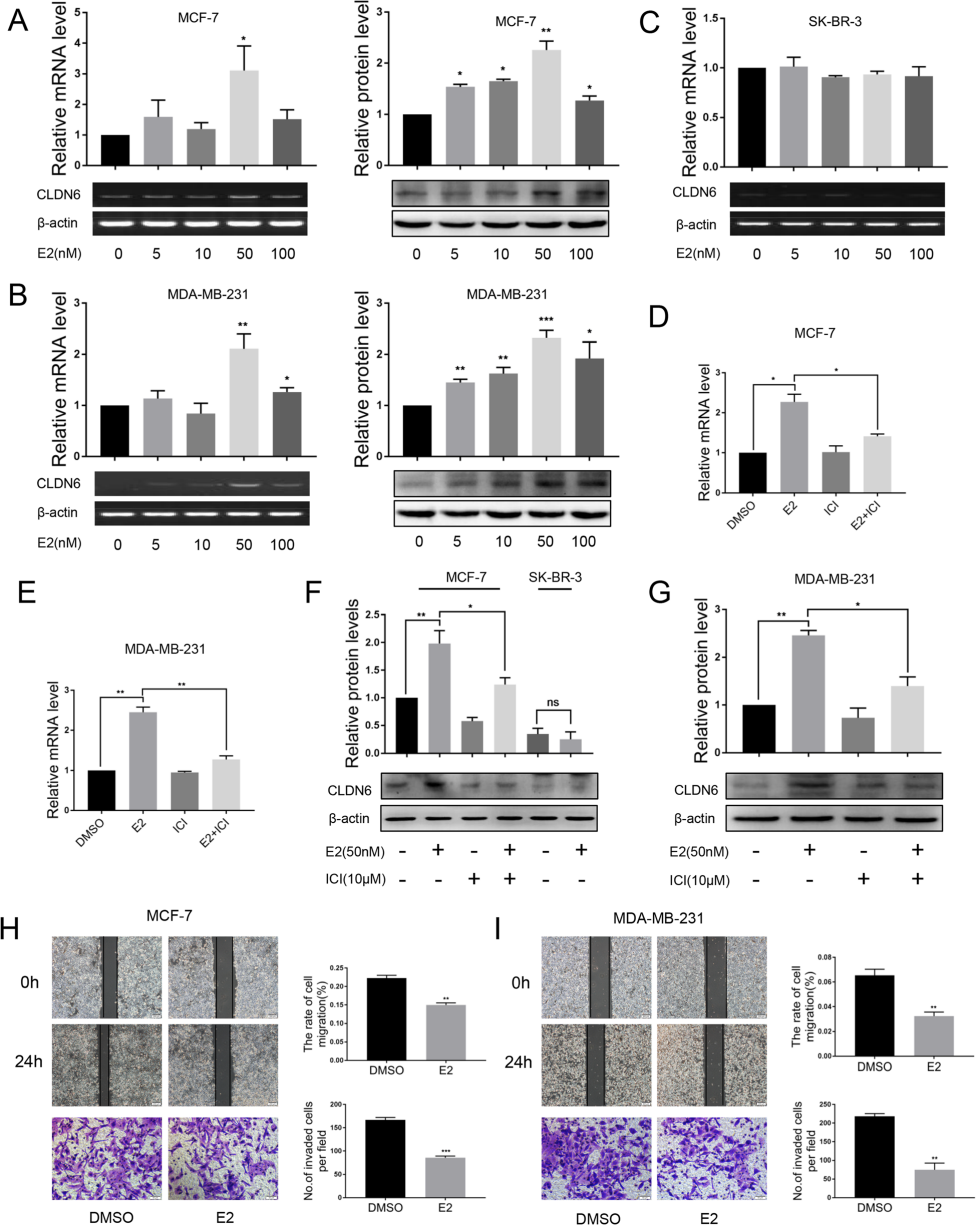
E2 upregulates the expression of CLDN6 in MCF-7 and MDA-MB-231 cells. MCF-7(**a**), MDA-MB-231(**b**) and SK-BR-3(**c**) cells were treated with DMSO or E2 (from 5 nM to 100 nM). Cells were harvested after 24 h treatment for analysis of gene and protein expression of CLDN6 using semiquantitative RT-PCR and western blot, respectively. Actin served as a loading control. Quantification of CLDN6 expression in MCF-7 (**d**) and MDA-MB-231 (**e**) cells cotreated with ICI by qRT-PCR. CLDN6 protein expression levels in MCF-7 (**f**) and MDA-MB-231 (**g**) cells after treatment with E2 or E2/ICI. CLDN6 protein expression in SK-BR-3 (**f**) cells after 50 nM E2 treatment. Wound healing and Transwell assays were used to detect the migration (scale bar, 200 μm) and invasion (scale bar, 50 μm) abilities of MCF-7 (**h**) and MDA-MB-231 (**i**) cells treated with E2. Data are presented as mean ± SD. The data shown are representative results of three independent experiments. *P < 0.05, **P < 0.01, ***P < 0.001

Estrogen signaling pathways are classified as genomic and nongenomic. Genomic pathways depend on transcriptional modulation of target genes by ERs, and nongenomic pathways mediate rapid activation of signaling cascades partly via membrane-bound G protein coupled estrogen receptor 1 (GPER1/GPR30) [27–30]. To evaluate a potential functional link between E2 and CLDN6 in breast cancer cells, we treated MCF-7 (ERα+/ERβ+/GPR30+) and MDA-MB-231 (ERα−/ERβ+/GPR30-) cells with DMSO or E2 (from 5 nM to 100 nM) for 24 h. The expression of CLDN6 was measured using semiquantitative RT-PCR and western blot. E2 treatment significantly upregulated the mRNA and protein expression of CLDN6 in a dose-dependent manner in MCF-7 cells, and 50 nM E2 showed the highest upregulation (Fig. 1a). Similar results were observed in MDA-MB-231 cells (Fig. 1b). However, the E2-induced effect on CLDN6 was not observed at the mRNA level in SK-BR-3 (ERα−/ERβ−/GPR30+) cells (Fig. 1c), and 50 nM E2 did not exert significant effects on CLDN6 protein levels in SK-BR-3 cells (Fig. 1f). The results indicated that E2 did not regulate CLDN6 through GPR30. We treated MCF-7 and MDA-MB-231 cells with a nonselective estrogen receptor antagonist ICI 182.780 (ICI). ICI cotreatment counteracted the E2-induced effects on CLDN6 mRNA and protein levels (Fig. 1 d-g), suggesting a direct involvement of the ERs in the regulation of CLDN6. Since we have previously demonstrated that CLDN6 overexpression led to a lower migration and invasion of breast cancer cells [20, 22], we investigated whether E2-induced CLDN6 expression was involved in reducing breast cancer cell migration and invasion. In agreement with previous studies, E2 treatment caused a reduction of migration and invasion in MCF-7 cells as well as in MDA-MB-231 cells (Fig. 1 h, i).

### E2 regulates CLDN6 expression through ERβ

**Fig. 2:**
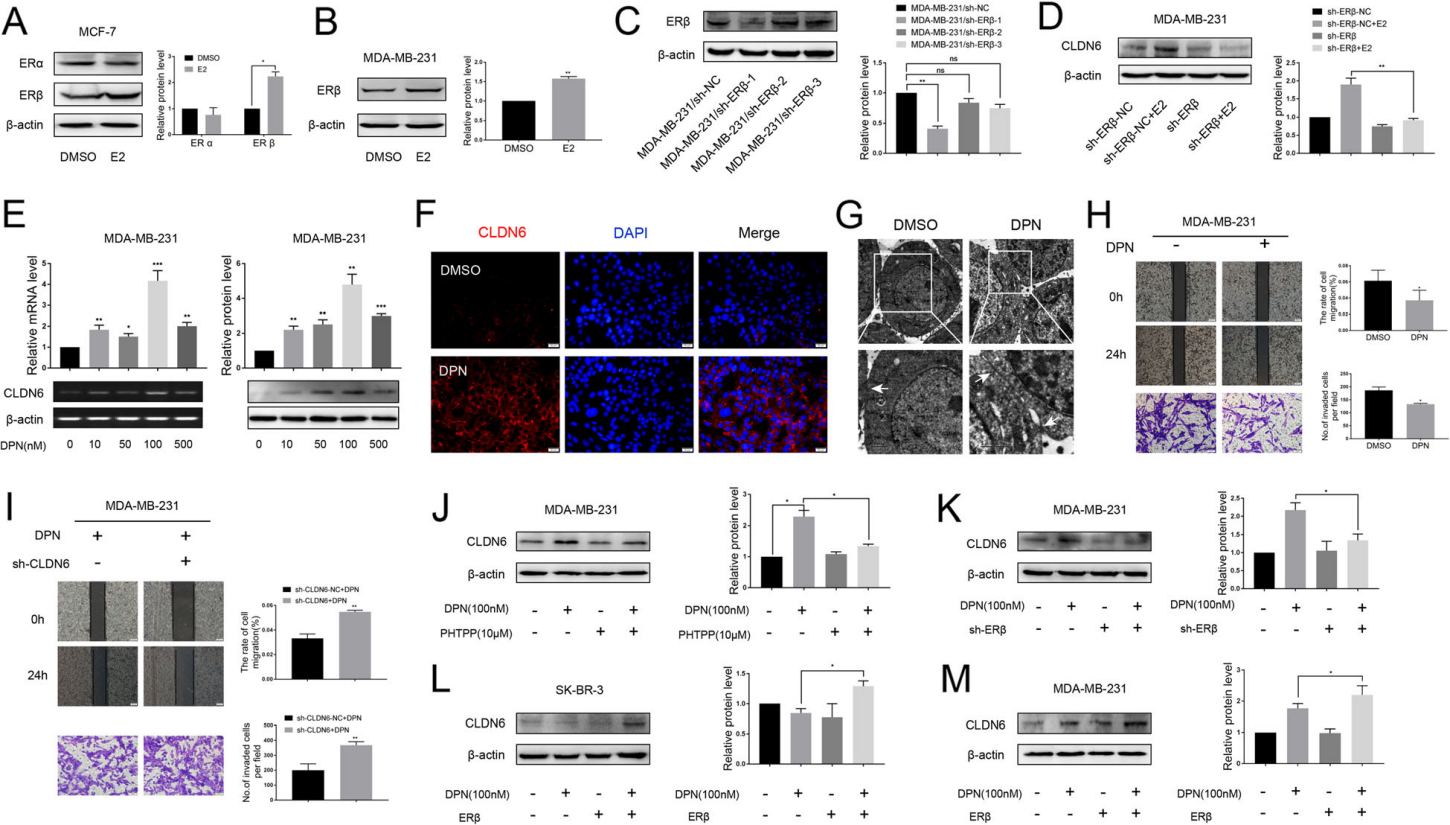
E2 regulates CLDN6 expression via ERβ. **a** Western blot analysis of ERα and ERβ expression in MCF-7 cells treated with E2. Actin served as a loading control. **b** Western blot analysis of ERβ in MDA-MB-231 cells treated with E2. **c** MDA-MB-231 cells were transfected with either negative control (sh-NC) or three different ERβ shRNAs for 48 h and were then subjected to western blot analysis to detect the protein abundance of ERβ. Actin was used as the loading control. **d** ERβ knockdown abolished the CLDN6 expression induced by E2. **e** MDA-MB-231 cells were incubated with DPN for 24 h at the indicated concentration. CLDN6 gene and protein expression levels were detected by using semiquantitative RT-PCR and western blot. **f** Immunofluorescence of CLDN6 (red) was prominent along the edges of the MDA-MB-231 cells upon DPN treatment. Nuclei were stained with 4, 6-diamino-2-phenylindole (DAPI) (blue) (scale bar, 20 μm). **g** Tight junctions (white arrowheads) between cells were prominent in MDA-MB-231 cells after DPN treatment as observed by TEM. **h** The migration (scale bar, 200 μm) and invasion (scale bar, 50 μm) abilities of MDA-MB-231 cells treated with DPN were decreased. **i** CLDN6 knockdown rescued the migration and invasion abilities of MDA-MB-231 cells after DPN treatment. The ERβ antagonist PHTPP (10 μM, 24 h) (**j**) and ERβ knockdown (**k**) abolished the DPN-induced CLDN6 expression. Overexpression of ERβ induced CLDN6 upregulation in SR-BR-3 (**l**) and MDA-MB-231 (**m**) cells after treatment with DPN. Data are presented as mean ± SD. The data shown are representative results of three independent experiments. *P < 0.05, **P < 0.01, ***P < 0.001

In this study, we found that the expression of CLDN6 was enhanced in MCF-7 and MDA-MB-231 cells by E2. Moreover, when MCF-7 and MDA-MB-231 cells were treated with 50 nM E2, the expression of ERβ in both cells was increased, but ERα expression was not changed in MCF-7 cells (Fig. 2 a, b). Therefore, this E2-induced effect was not ERα-dependent, and we focused our attention on ERβ. To demonstrate that the increased expression of CLDN6 was mediated by ERβ, we knocked down ERβ in MDA-MB-231 cells. Three different ERβ short hairpin RNAs (shRNAs) were tested, and ERβ shRNA-1 was used in the following experiments (Fig. 2c). The depletion of ERβ resulted in no increase in CLDN6 expression after E2 treatment in MDA-MB-231 cells (Fig. 2d). Thus, the induction effect of E2 on CLDN6 could be achieved through ERβ. To observe the regulatory role of ERβ on CLDN6, MDA-MB-231 cells were treated with various concentrations of diarylpropionitrile (DPN), a selective ERβ agonist. It was found that the effect of DPN on CLDN6 expression was similar to that of E2, and the most effective concentration of DPN was 100 nM (Fig. 2e). The immunoreactivity of CLDN6 was prominent along the edges of the MDA-MB-231 cells upon DPN treatment as demonstrated by immunofluorescence (Fig. 2f). By TEM we observed that the tight junctions between cells were prominent in DPN treated MDA-MB-231 cells (Fig. 2g). Moreover, the migration and invasion abilities of MDA-MB-231 cells treated with DPN were decreased, which was consistent with the effect of E2 (Fig. 2h). Next, we asked whether the inhibitory effect of ERβ on migration and invasion of breast cancer cells was associated with CLDN6. We measured the effects of CLDN6 knockdown on the migration and invasion of MDA-MB-231 cells treated with DPN. The results showed that the DPN-induced reduction in migration and invasion could be rescued by the transfection of CLDN6 shRNA in MDA-MB-231 cells (Fig. 2i). CLDN6 knockdown efficiency was detected via western blot (Additional file 1: Figure S1A). To further explore the regulation of ERβ on CLDN6, we used PHTPP (a selective ERβ antagonist) and ERβ shRNA. The results showed that depletion of ERβ abolished the DPN-induced CLDN6 expression in MDA-MB-231 cells (Fig. 2 j, k). Conversely, overexpression of ERβ induced CLDN6 upregulation in SR-BR-3 and MDA-MB-231 cells after treatment with DPN (Fig. 2l, m). We also observed that the depletion or upregulation of ERβ did not affect the expression of CLDN6 in the absence of DPN (Fig. 2j-m). ERβ transfection efficiency was detected via western blot (Additional file 1: Figure S1B). These results suggest that ERβ regulates CLDN6 expression in a ligand-dependent manner.

### ERβ regulates CLDN6 expression at the transcriptional level

**Fig. 3:**
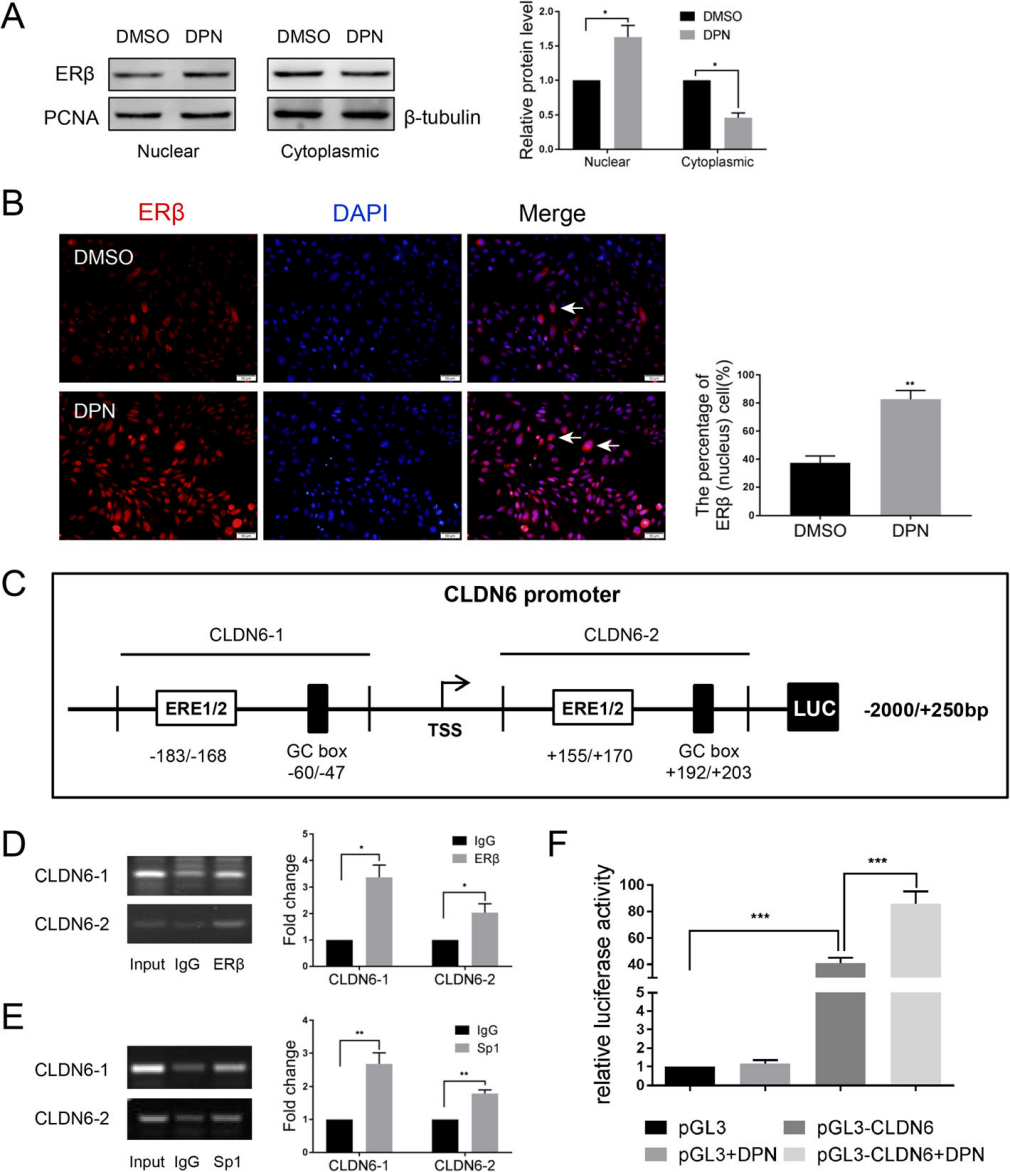
ERβ regulates CLDN6 expression at the transcriptional level. **a** ERβ protein expression in the nuclear and cytoplasmic fractions of DPN-treated MDA-MB-231 cells. PCNA served as a nuclear protein control, and β-tubulin was used as a cytoplasmic protein control. **b** Subcellular localization of ERβ. DPN promotes the transportation of ERβ from the cytoplasm to the nucleus (white arrowheads) in MDA-MB-231 cells. Nuclei were stained with DAPI (blue) (scale bar, 50 μm). **c** Schematic diagram of the sequence spanning from -2000 bp to + 250 bp relative to the translational start site (TSS) of the CLDN6 gene promoter containing the (half-ERE)-(N)x-(GC-box) motif. **d** ChIP assay. The ERβ binding sites (− 183/− 168 bp or + 155/+ 170 bp) of the CLDN6 promoter were detected by PCR in DPN-treated MDA-MB-231 cells. **e** ChIP assay. The Sp1 binding sites (− 60/− 47 bp or + 192/+ 203 bp) of the CLDN6 promoter were detected by PCR in DPN-treated MDA-MB-231 cells. **f** The luciferase activities of DPN-treated MDA-MB-231 cells transfected with pGL3-CLDN6 and renilla luciferase reporter (pRL-TK) plasmids were detected by dual-luciferase reporter assays. Data are presented as mean ± SD. The data shown are representative results of three independent experiments. *P < 0.05, **P < 0.01, ***P < 0.001

In the genomic pathway, ERs act as transcription factors and regulate the transcription of target genes directly by binding to estrogen response elements (EREs) in the promoter region of genes or indirectly by interacting with other transcription factors, such as stimulating protein 1 (Sp1) and activating protein-1 (AP1) [27, 29]. Nuclear and cytoplasmic proteins were extracted and examined for ERβ expression. Our results demonstrated that nuclear ERβ protein was increased, whereas cytoplasmic ERβ protein was decreased in DPN treated MDA-MB-231 cells (Fig. 3a). Immunofluorescence experiments showed that DPN induced the translocation of ERβ from the cytoplasm to the nucleus in MDA-MB-231 cells (Fig. 3b). Next, we analyzed the CLDN6 promoter sequence through the UCSC (http://genome.ucsc.edu/) and JASPAR databases (http://jaspardev.genereg.net/). In the present investigation, we were not able to identify a conventional ERE [31], 5′-GGTCAnnnTGACC-3′, in the CLDN6 promoter. However, we predicted potential ERβ-binding sites (half-ERE) and Sp1 binding sites (GC-box) through bioinformatics analysis in JASPAR. To examine whether ERβ or Sp1 could bind to the CLDN6 promoter region in cells, we performed ChIP assays. The primers for the ChIP assays included the potential half-ERE and Sp1 binding sites (Fig. 3c). The results indicated that ERβ and Sp1 bound to the CLDN6 promoter in DPN-treated cells (Fig. 3d, e). To further confirm that ERβ regulated CLDN6 promoter activity, we performed dual luciferase reporter assays. The CLDN6 promoter fragment − 2000/+ 250 bp was cloned into the pGL3-basic vector and then cotransfected with renilla luciferase reporter plasmid into MDA-MB-231 cells (Fig. 3c). Both ERβ and Sp1 are expressed in MDA-MB-231cells. After transfection, the cells were treated with 100 nM DPN for 24 h. We found that luciferase activity was significantly increased in DPN-treated pGL3-CLDN6 cells compared with the pGL3-CLDN6 cells (Fig. 3f). These data provide evidence that ERβ regulates CLDN6 at the transcriptional level.

### ERβ induces autophagy and suppresses the migration and invasion of breast cancer cells

**Fig. 4:**
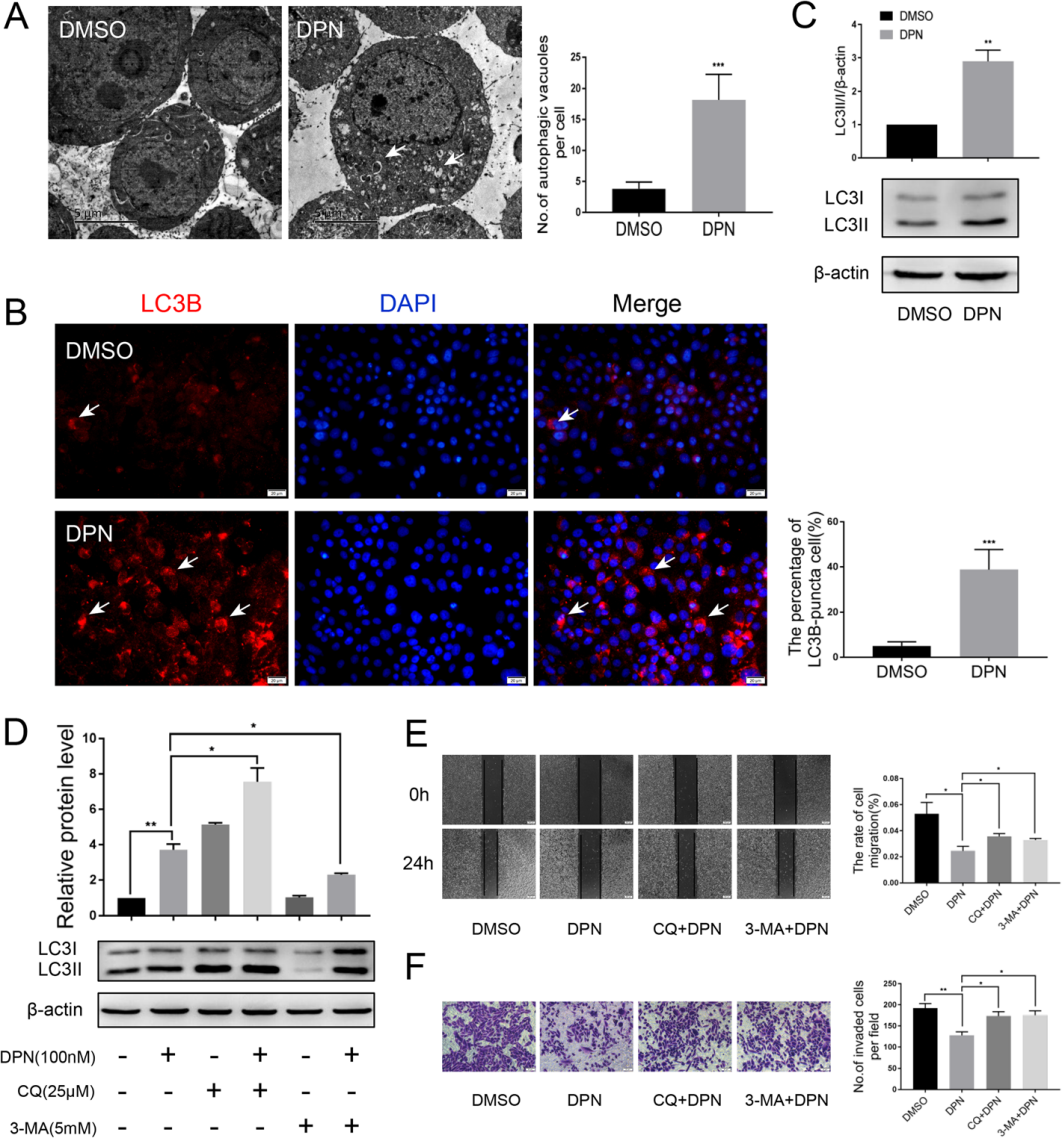
ERβ induces autophagy and suppresses the migration and invasion of breast cancer cells. **a** TEM analysis was performed on MDA-MB-231 cells treated or not treated for 24 h with 100 nM DPN. DPN-treated cells displayed several autophagic vacuoles with the characteristic double membrane (white arrowheads) which were not observed in the control (scale bar, 5 μm). **b** Immunofluorescence analysis showed that the numbers of LC3-II puncta (white arrowheads) were increased after DPN treatment. Nuclei were stained with DAPI (Scale bar, 20 μm). **c** The expression of LC3B was detected by western blot in DPN-treated or untreated MDA-MB-231 cells. Actin served as a loading control. **d** The expression of LC3B was detected by western blot in MDA-MB-231 cells cotreated with DPN and CQ (25 μM, 24 h) or DPN and 3-MA (5 mM, 24 h). CQ and 3-MA were pretreated for 2 h before treatment with DPN. Wound healing (**e**) and Transwell migration (**f**) assays showed that the DPN-inhibited migration (scale bar, 200 μm) and invasion (scale bar, 50 μm) abilities were attenuated by CQ and 3-MA. CQ and 3-MA were pretreated for 2 h before treatment with DPN. Data are presented as mean ± SD. The data shown are representative results of three independent experiments. *P < 0.05, **P < 0.01, ***P < 0.001

Previously, we observed the ultrastructure of MDA-MB-231 cells treated with DPN by TEM. Surprisingly, we found that a large number of autophagic vacuoles appeared in the cytoplasm of DPN-treated cells compared with control cells (Fig. 4a). It is known that the amount of LC3B-II represents the number of autophagosomes [32, 33]. In this study, LC3B-II-labeled puncta formation was also observed through fluorescence microscopy in DPN-treated cells, but fewer puncta appeared in untreated cells (Fig. 4b). We analyzed the protein expression level of LC3B by western blot, and we found that the ratio of LC3-II/I was significantly increased in MDA-MB-231 cells treated with DPN (Fig. 4c). To further prove that DPN-activated ERβ induces autophagy, we detected LC3B expression in MDA-MB-231 cells with ERβ knockdown after DPN treatment. The western blot results showed that with ERβ knocked down, DPN did not induce an increase in the LC3-II/I ratio in MDA-MB-231 cells (Additional file 2: Figure S2A). Immunofluorescence assays showed that LC3B-II-labeled puncta were rarely observed in DPN-treated ERβ-knockdown cells (Additional file 2: Figure S2B). These results suggest that ERβ might be involved in the induction of autophagy. In fact, LC3-II can accumulate due to increased autophagosome formation or a block in the autophagosome-lysosome fusion process. To distinguish between these two possibilities, we assayed the LC3-II level in the presence of chloroquine (CQ), which blocks autophagosome-lysosome fusion and leads to the accumulation of autophagosomes. After treatment with DPN and CQ, the ratio of LC3-II/I was increased 2.5 times compared with that in MDA-MB-231 cells treated with DPN alone (Fig. 4d). Moreover, when MDA-MB-231 cells were treated with DPN and 3-methyladenine (3-MA) (an inhibitor of early phases of autophagy), the DPN-induced increase was dramatically reduced (Fig. 4d). These results suggest that ERβ is involved in the early steps of autophagosome formation. To understand the biological significance of ERβ-induced autophagy, we tested the migration and invasion of breast cancer cells treated with autophagy inhibitors. We found that both CQ and 3-MA effectively hindered the effects of DPN on migration and invasion (Fig. 4e, f).

### ERβ induces autophagy via the CLDN6-mediated increase in beclin1

**Fig. 5:**
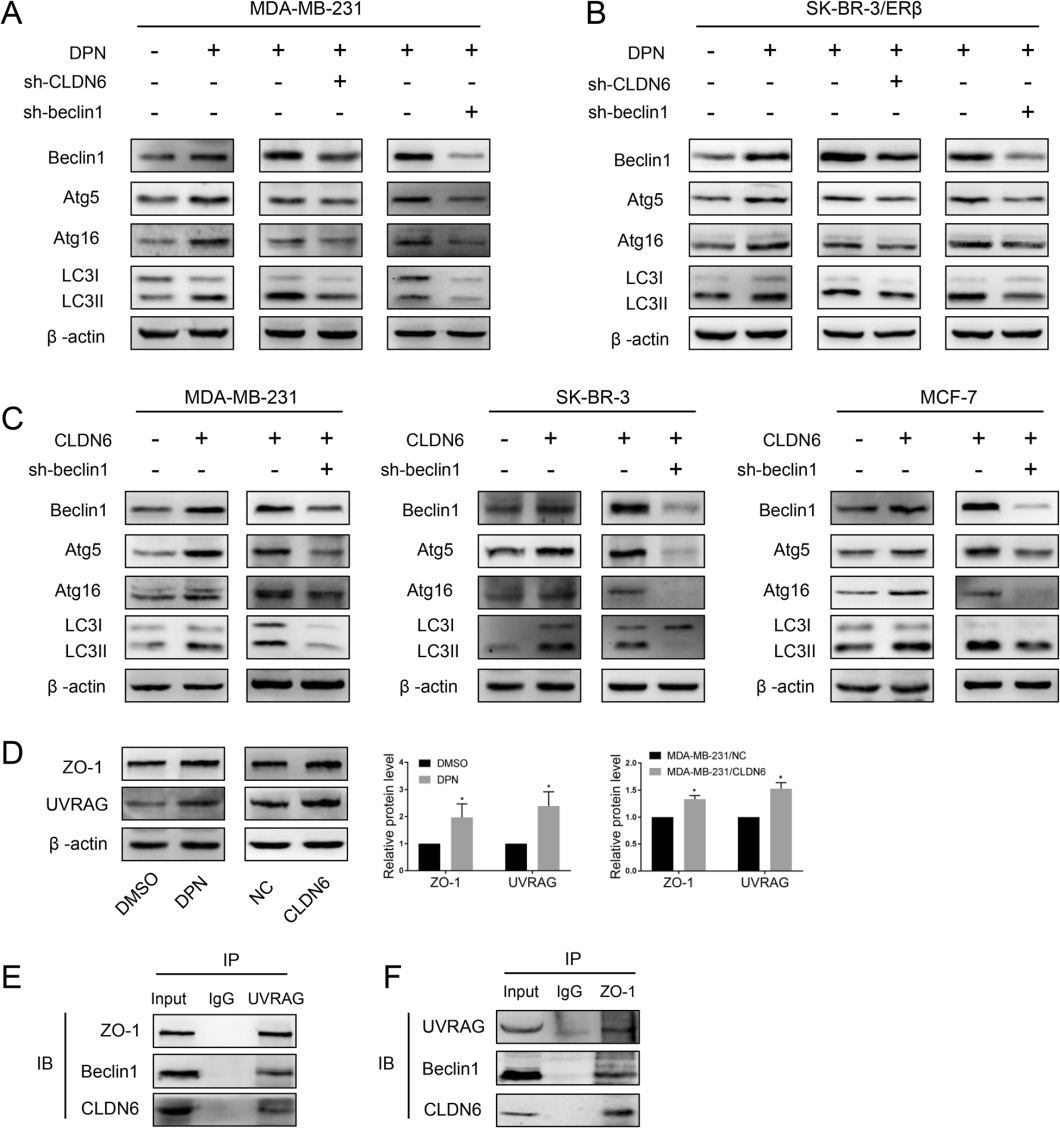
ERβ induces autophagy via CLDN6-mediated increase in beclin1. Expression of autophagy-related proteins was detected by using western blot in DPN-treated cells and in CLDN6-knockdown and beclin1-knockdown DPN-treated MDA-MB-231 (**a**) and SK-BR-3/ERβ (**b**) cells. Actin served as a loading control. **c** Expression of autophagy-related proteins was detected by using western blot in CLDN6-overexpressing MDA-MB-231, SK-BR-3 and MCF-7 cells and CLDN6-overexpressing beclin1-knockdown cells. **d** Western blot analysis of ZO-1 and UVRAG in DPN-treated and CLDN6-overexpressing MDA-MB-231 cells. **e-f** Co-IP assays were performed to detect the interaction between beclin1 and CLDN6 in MDA-MB-231 cells treated with DPN. Data are presented as mean ± SD. The data shown are representative results of three independent experiments. *P < 0.05

To identify the mechanisms underlying the ERβ activation-induced effects described above, we used western blot to detect biomarkers of the early stage of autophagy formation. The results showed that beclin1, atg5, atg16 and LC3-II were significantly increased in MDA-MB-231 and SK-BR-3/ERβ (ERβ-overexpressing SK-BR-3 cells) cells after DPN treatment (Fig. 5a, b). The expression of CLDN6 is shown in Additional file 3: Figure S3A. In our previous work, we found that CLDN6 induced autophagy in MCF-7 cells. Thus, we wanted to detect the relationship between CLDN6 and ERβ-induced autophagy. We knocked down CLDN6 in DPN-treated cells, and western blot analyses showed that beclin1, atg5, atg16 and LC3-II expression was decreased (Fig. 5a, b). The expression of CLDN6 is shown in Additional file 3: Figure S3A. These results suggested that ERβ induced autophagy via the regulation of CLDN6. Interestingly, the expression of beclin1, atg5, atg16 and LC3-II proteins was also increased in MDA-MB-231/CLDN6, SK-BR-3/CLDN6 and MCF-7/CLDN6 cells (CLDN6-overexpressing MDA-MB-231, SK-BR-3 and MCF-7 cells) (Fig. 5c). The expression of CLDN6 is shown in Additional file 3: Figure S3B. Given that CLDN6 is strongly associated with autophagy in breast cancer cells, we wanted to explore the role of CLDN6 in autophagy. The above results showed that CLDN6 overexpression or CLDN6 knockdown led to an increase or decrease in beclin1 expression, respectively (Fig. 5a, b). The key autophagy regulator beclin1 might be a downstream molecule of CLDN6. Hence, we presumed that CLDN6 regulated autophagy through beclin1. To verify this hypothesis, we depleted beclin1 in DPN-treated MDA-MB-231 and SK-BR-3/ERβ cells by using lentiviral vectors. Beclin1 knockdown efficiency was detected via western blot. We found that the expression levels of atg5, atg16 and LC3-II were significantly decreased in both cells (Fig. 5a, b). Similar regulatory roles of beclin1 on autophagy were confirmed in MDA-MB-231/CLDN6, SK-BR3/CLDN6 and MCF-7/CLDN6 cells (Fig. 5c). Taken together, these results showed that the regulation of CLDN6 on autophagy was beclin1-dependent. Next, we addressed the question of how does CLDN6 regulate beclin1. It is noteworthy that UV-radiation resistance associated gene (UVRAG) is a positive regulator of beclin1 [34]. UVRAG forms a complex with beclin1 and is involved in autophagosome formation [34]. Theoretically, UVRAG can bind with proteins containing Src homology (SH3) domains [35]. Interestingly, ZO-1 contains a C-terminal SH3 domain [36]. ZO-1 is a scaffold protein that directly binds to PDZ motifs on the extreme C-terminus of CLDNs [37]. Thus, we speculated that ZO-1 and UVRAG might function as bridge molecules for the CLDN6-beclin1 interaction. Western blot analyses showed that the expression levels of ZO-1 and UVRAG were increased in both DPN-treated and CLDN6-overexpressing cells (Fig. 5d). Because CLDN6 expression of MDA-MB-231 cells was low, we used DPN-treated cells to perform co-IP assays. The results revealed that ZO-1 and UVRAG indeed had binding affinities for CLDN6 and beclin1 in DPN-treated cells (Fig. 5e, f). These results indicated that CLDN6 and ZO-1/UVRAG/beclin1 formed complexes with other autophagy proteins to regulate autophagosome formation in breast cancer cells.

### CLDN6 inhibits migration, invasion and metastasis of breast cancer through beclin1 in vitro and in vivo

**Fig. 6:**
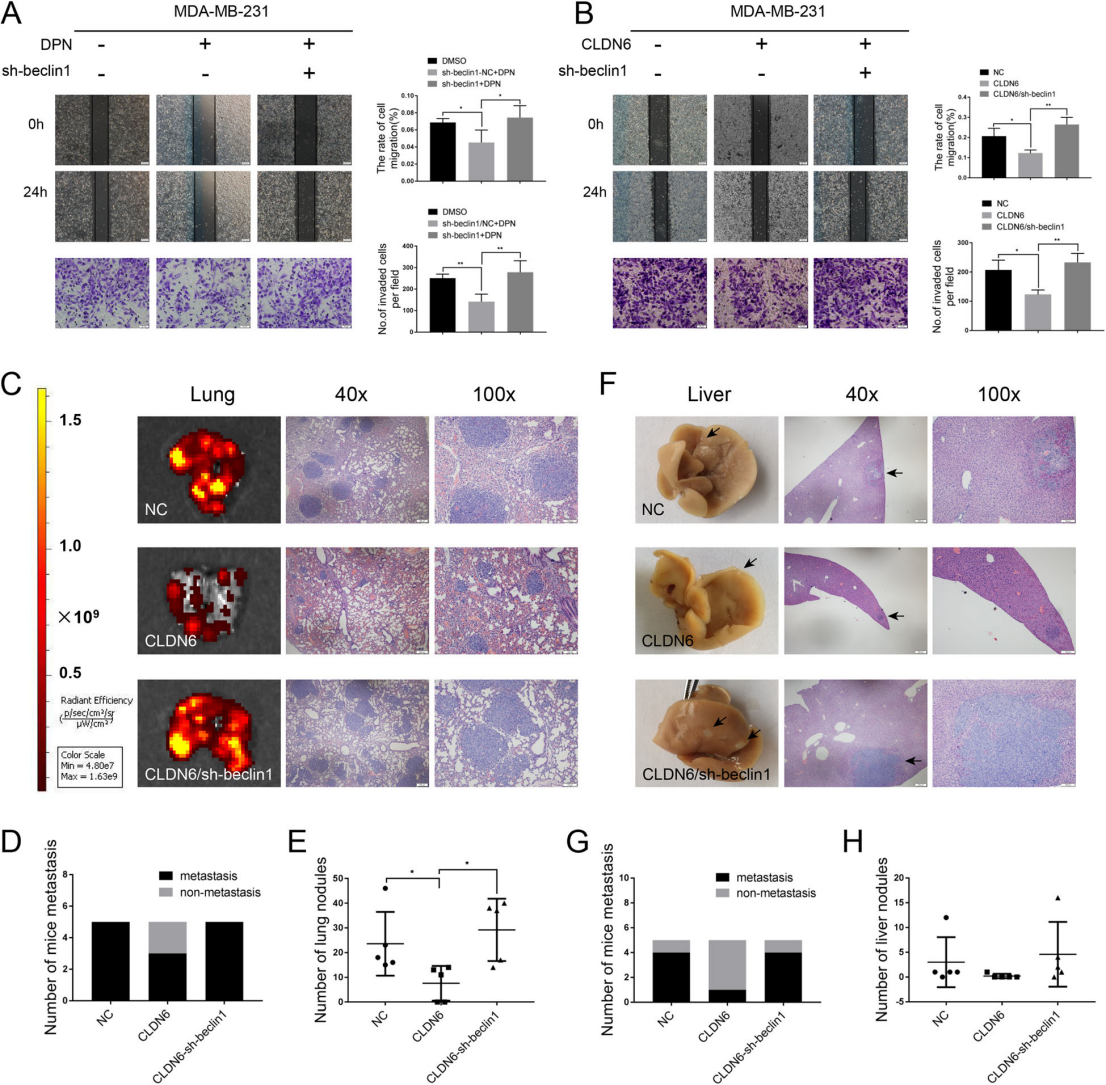
CLDN6 inhibits migration, invasion and metastasis of breast cancer through beclin1 in vitro and in vivo. Wound healing and Transwell migration assays showed that beclin1 knockdown drove the migration (scale bar, 200 μm) and invasion (scale bar, 50 μm) of DPN-treated (**a**) and CLDN6-overexpressing MDA-MB-231 cells (**b**). **c** IVIS imaging was performed to show lung metastatic sites. Representative H&E staining of lung sections from the three groups (*n* = 5). **d** The number of mice with lung metastasis was counted in each group. **e** The number of lung nodules in each group. **f** Representative images of liver metastasis and H&E staining in the three groups (*n* = 5). Black arrowheads indicate the metastatic sites. **g** The number of mice with liver metastasis was counted in each group. **h** The number of liver nodules in each group. Data are presented as mean ± SD. The data shown are representative results of three independent experiments. *P < 0.05, **P < 0.01

When beclin1 in DPN-treated MDA-MB-231 (DPN-induced CLDN6 expression) cells and MDA-MB-231/CLDN6 (overexpressed exogenous CLDN6) cells was silenced, the migration and invasion abilities of both cells increased, and the inhibitory effects of CLDN6 were abrogated (Fig. 6a, b). The results suggested that CLDN6 inhibited the migration and invasion of breast cancer cells in vitro through beclin1.

To further investigate the anti-metastatic effect of CLDN6 in vivo, we established lung metastasis mouse models using MDA-MB-231/NC cells (control MDA-MB-231 cells), MDA-MB-231/CLDN6 cells (CLDN6-overexpressing MDA-MB-231 cells) and MDA-MB-231/CLDN6-sh-beclin1 cells (CLDN6-overexpressing and beclin1-knockdown MDA-MB-231 cells). Lung metastasis was determined by a comprehensive analysis of macroscopic observations and hematoxylin and eosin (H&E) staining of lung tissue sections. The results showed that there was a higher occurrence of lung metastasis in the MDA-MB-231/NC and MDA-MB-231/CLDN6-sh-beclin1 groups and a lower occurrence of lung metastasis in MDA-MB-231/CLDN6 group (Fig. 6c-e). Figure 6c shows the representative H&E staining of lung tissues from mice in the three groups. Surprisingly, we also observed liver metastasis in the three groups. There were 4 cases of liver metastasis in both the MDA-MB-231/NC and MDA-MB-231/CLDN6-sh-beclin1 groups. However, 1 case was found in the MDA-MB-231/CLDN6 group (Fig. 6f-h). Our results showed that CLDN6 inhibited breast cancer metastasis by beclin1 in vivo.

### The clinical correlation analyses of ERβ, CLDN6 and beclin1 expression and prognosis in breast cancer patients

**Fig. 7:**
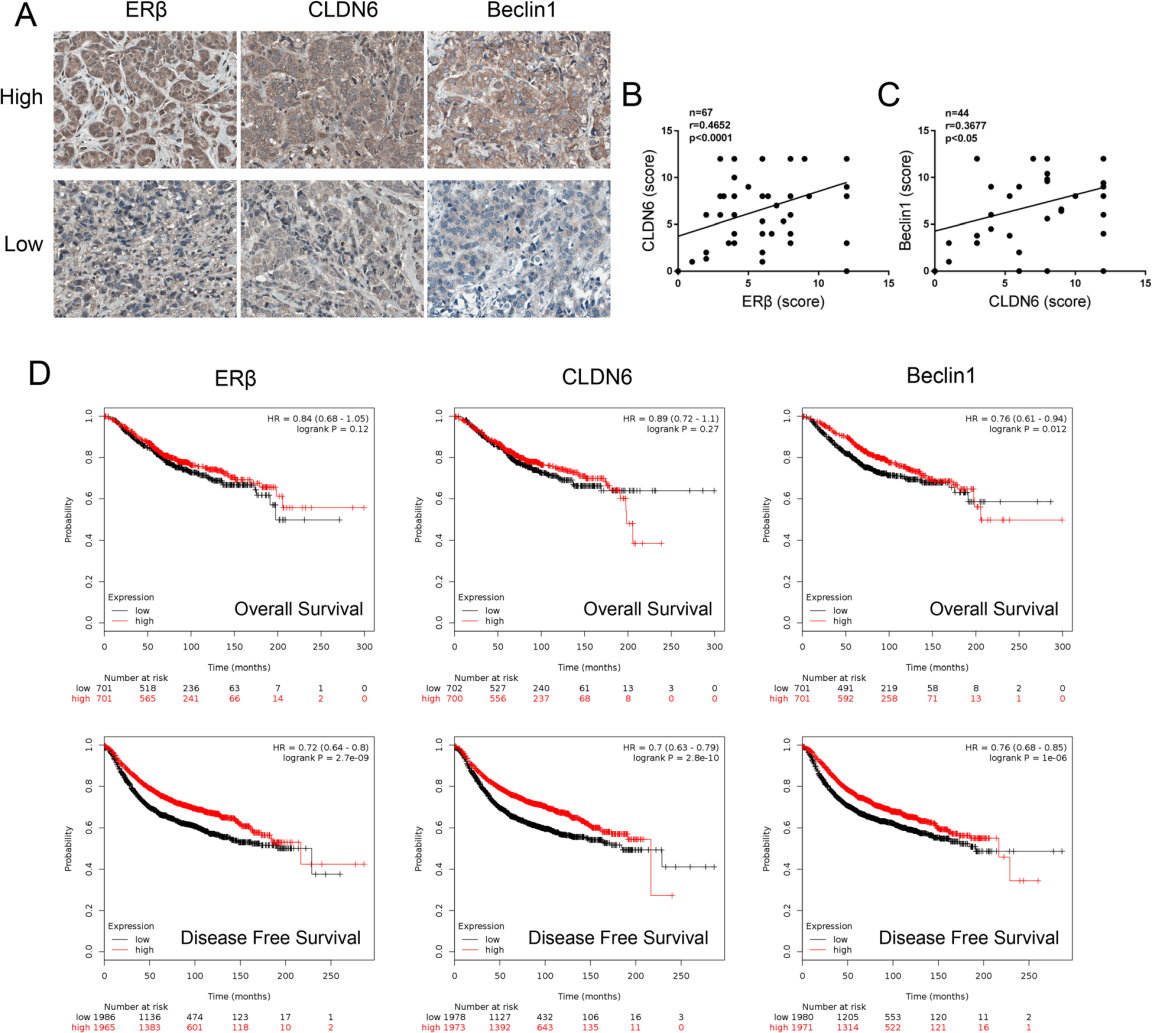
Clinical correlation analyses between ERβ, CLDN6 and beclin1 expression and prognosis in breast cancer patients. **a** Representative images of IHC analysis of ERβ, CLDN6 and beclin1 expression from tissue microarray (× 400). In breast cancer tissues and adjacent tissues, ERβ expression was observed in the nucleus and the cytoplasm, CLDN6 was expressed in the cytoplasm and the plasma membrane and beclin1 was mainly expressed in the cytoplasm. “Low” indicates proteins with low expression, and “high” indicates proteins with high expression. **b** Correlation between ERβ expression and CLDN6 expression in breast cancer tissue microarray. Pearson correlation test, *n* = 67, r = 0.4652, *P* < 0.0001. **c** Correlation between CLDN6 expression and beclin1 expression in breast cancer tissue microarray. Pearson correlation test, *n* = 44, r = 0.3677, *P* = 0.0141. **d** Kaplan-Meier analysis of the overall survival and disease-free survival of breast cancer patients with different ERβ, CLDN6 and beclin1 expression levels in the in Kaplan-Meier plotter database. Statistical differences were determined by log-rank test. Data are presented as mean ± SD

We next evaluated the expression of ERβ, CLDN6 and beclin1 in tumor samples from 70 breast cancer patients by immunohistochemical (IHC) staining of the tissue microarray. The expression of ERβ, CLDN6 and beclin1 in each IHC sample was classified as either high (score > 8) or low (score ≤ 8), using the median of the IHC scores as the cut-off value. The relationship between ERβ, CLDN6 and beclin1 expression and clinicopathologic features was further evaluated and the results are summarized in Table 1. We found that high expression of beclin1 was significantly associated with smaller tumors (*P* = 0.026) and that low expression of ERβ was significantly associated with early TNM stage (TNM, *P* = 0.023). No association existed between the expression of ERβ, CLDN6 and beclin1 and patient age, lymph node metastasis or pathological grade. Figure 7a shows the representative IHC staining of ERβ, CLDN6 and beclin1 in breast tissues. Pearson correlation analyses indicated that ERβ and CLDN6 were positively correlated and that the expression of CLDN6 was positively correlated with beclin1 in breast cancer tissues (Fig. 7b-c). There was no correlation between the expression of ERβ and beclin1 (*P* = 0.243). In addition, we further assessed the correlation between ERβ, CLDN6 and beclin1 expression and the overall survival (OS) and disease free survival (DFS) of breast cancer patients in the Kaplan-Meier plotter database (http://www.kmplot.com) by Kaplan-Meier analyses. The results showed that breast cancer patients with high beclin1 expression had significantly longer OS than patients with low beclin1 expression, and patients with high ERβ, CLDN6 and beclin1 expression levels showed longer DFS than the respective low expression groups (Fig. 7d). Taken together, these findings suggest that high expression of ERβ, CLDN6 or beclin1 indicates a better prognosis for breast cancer patients.

**Table 1:** The association between ERβ/ CLDN6/ beclin1 expression and clinical characteristics of breast cancer patients

Characteristic	ERβ		*p*	CLDN6		*p*	Beclin1		*p*
	High	Low		High	Low		High	Low	
Age			0.932			0.923			0.697
≤57 years	11	22		13	20		8	11	
>57 years	11	23		13	21		12	13	
Pathology grade			0.785			0.717			0.960
I-II	10	19		11	19		7	11	
II-III	11	18		9	19		8	13	
Tumour size			0.451			0.716			0.026
≤3 cm	13	29		16	25		15	10	
>3 cm	10	15		9	17		5	14	
Lymph node			0.314			0.088			0.899
Negative	8	24		12	20		11	14	
Positive	9	15		4	20		6	7	
TNM stage			0.023			0.356			0.947
0-II	13	37		20	30		14	20	
III-IV	10	8		5	13		4	6

## Discussion

Our previous studies have shown that E2 upregulated CLDN6 expression and hindered MCF-7 cell migration and invasion, but the molecular mechanisms were still unclear. In this study, we found that the regulation of CLDN6 by E2 and its biological effects were mediated by ERβ and were not ERα-dependent. Furthermore, the inhibitory effects of ERβ on the migration and invasion of breast cancer cells were mediated through CLDN6-induced autophagy. To our knowledge, this is the first study to link the inhibitory role of ERβ on migration and invasion and autophagy modulation in breast cancer cells.

Estrogen has pivotal roles in the development and progression of breast cancer. The biological effects of estrogen are mediated by intracellular ERs (ERα and ERβ) and the cell-membrane receptor GPR30. GPR30 is a member of the G-protein coupled receptor (GPCR) superfamily. Estrogen mediates rapid nongenomic actions and cell biological functions partly through GPR30 [38, 39]. Our study found that E2 induced the expression of CLDN6 in MCF-7 (ERα+/ERβ+/GPR30+) and MDA-MB-231 (ERα−/ERβ+/GPR30-) cells but not in SK-BR-3 (ERα−/ERβ−/GPR30+) cells. Moreover, ICI (a nonselective estrogen receptor antagonist) cotreatment counteracted the E2-induced effects. These results indicated that the regulation of E2 on CLDN6 was not through GPR30. ERα is a key target of endocrine therapy and induces proto-oncogene expression to stimulate cell proliferation in breast cancer [40]. Recently, a few studies have reported that ERα suppresses the migration and invasion of breast cancer cells by upregulating cytoskeleton protein expression [41–43]. Our results showed that the expression of CLDN6 was enhanced and migration and invasion were hindered in both MCF-7 and MDA-MB-231 cells treated with E2. Therefore, this E2-induced effect was not ERα-dependent, and we wanted to explore the role of ERβ in this process. The role of ERβ in cancer cells is still poorly understood. Growing evidence supports that ERβ is a tumor suppressor. ERβ has been shown to increase integrin α1β1 levels and inhibit the migration of breast cancer cells [44]. In addition, ERβ decreased basal-like breast cancer cell invasion by promoting degradation of epidermal growth factor receptor (EGFR) [45]. Our study found that the expression of ERβ was increased in MCF-7 and MDA-MB-231 cells treated with E2. E2 did not induce CLDN6 expression in MDA-MB-231 cells with ERβ knockdown. DPN is a selective ERβ agonist. DPN-mediated ERβ activation could also upregulate CLDN6 expression and reduce migration and invasion of MDA-MB-231 cells. When ERβ was knocked down in breast cancer cells, the expression of CLDN6 was not increased after DPN treatment. The effect of DPN on CLDN6 was similar to that of E2 in breast cancer cells. These results indicated that ERβ played a pivotal role in the regulation of CLDN6 by E2 and its effect on biological behavior. Although many studies have supported the idea that ERβ expression inhibits the migration and invasion of breast cancer cells, Zoi et al. found that ERβ knockdown decreased the expression of matrix metalloproteinases (MMPs) and promoted mesenchymal-epithelial transition (MET) to suppress MDA-MB-231 cell migration and invasion [7]. Hence, the precise role of ERβ and its regulatory mechanism in breast cancer still need to be elucidated.

Our previous study demonstrated that CLDN6 is a breast cancer suppressor gene. CLDN6 overexpression suppressed the migration and invasion of breast cancer cells by reversing epithelial-mesenchymal transition (EMT) [20, 22, 46]. Then, we examined whether the ERβ-induced effect on biological behavior was related to CLDN6. Here, our results showed that CLDN6 knockdown rescued the effects of ERβ on the migration and invasion of MDA-MB-231 cells. Furthermore, we investigated the regulatory effect of ERβ on CLDN6. ERβ is a member of the nuclear receptor superfamily and functions as a hormone-dependent transcription factor [1]. The classical model of ERβ activation is that after ligand binding to the receptor, ligand-ER complexes directly bind to EREs in the promoter regions of target genes or indirectly interact with other transcription factors (Sp1 or AP1) to activate the transcription of target genes [28, 29]. When the ligand is absent, ERβ can still be activated by growth factor receptors (insulin-like growth factor 1 receptor (IGF1R) and EGFR), which can stimulate protein kinase cascades that phosphorylate and activate the transcriptional activity of ERβ [47, 48]. In this study, we found that the depletion or upregulation of ERβ did not affect the expression of CLDN6 in the absence of DPN. However, CLDN6 expression in breast cancer cells overexpressing ERβ was increased after DPN treatment. Therefore, we believe that the regulation of CLDN6 by ERβ is a ligand-dependent pathway in breast cancer cells. Burek et al. reported that E2-induced CLDN5 upregulation was mediated by binding to the EREs and Sp1 sites of the CLDN5 promoter in brain endothelial cells [49]. Furthermore, we analyzed the promoter region of CLDN6 and identified imperfect EREs and potential Sp1 transcription factor binding sites. ChIP assays showed that the regulation of the CLDN6 promoter could take place either directly by interaction with ERβ or indirectly by interaction with Sp1. Dual luciferase reporter assays showed that ERβ regulated CLDN6 promoter activity. The exact molecular mechanism and promoter elements responsible for ERβ regulation require further investigation. We report for the first time, to our knowledge, that the inhibitory effects of ERβ on migration and invasion are mediated by CLDN6 and that CLDN6 is a target gene of ERβ in breast cancer.

Autophagy is a basic catabolic process in which unnecessary or dysfunctional intracellular materials are degraded and recycled [50]. Autophagy is reported to mediate an oncosuppressive role in the tumor initiation step [51]. Surprisingly, our data indicated that ERβ induced autophagy in breast cancer cells. While previous studies have reported that ERβ triggers autophagy and inhibits cancer cell proliferation [12, 52], few studies have investigated the role of ERβ-induced autophagy on the migration and invasion of breast cancer cells. In our study, autophagy inhibitors reversed the inhibitory effect of ERβ on the migration and invasion of breast cancer cells. These results indicated that the inhibitory role of ERβ on the migration and invasion of breast cancer cells was associated with ERβ-induced autophagy. Furthermore, we found that ERβ induced autophagy through CLDN6. CLDN6 is one of the key proteins in the formation of tight junctions (TJs), and plays important roles in maintaining the epithelial barrier, polarity and signal delivery. There are few studies on the relationship between CLDNs and autophagy. Recently, it has been reported that the autophagosome marker Atg16L colocalizes with CLDN5 in endocytosed vesicles transported across the cells, indicating that the process of tight junction remodeling involves the regulation of autophagy [53]. Zhao Z. et al. discovered that CLDN1 regulated drug resistance by promoting autophagy, which was mediated by ULK1 phosphorylation in non-small cell lung cancer [54]. In addition, J. Kim et al. reported that CLDN1 functions as an autophagy stimulator to increase autophagy flux and accelerate the degradation of SQSTM1/p62 [55]. In our previous investigation, we observed by TEM that CLDN6 overexpression resulted in the appearance of numerous autophagic vacuoles. Western blot analysis and IF and acridine orange (AO) staining methods demonstrated that CLDN6 induced autophagy in breast cancer cells. Nevertheless, none of the above studies has clarified the regulatory mechanism of CLDNs on autophagy.

Next, we wanted to unveil the mechanism by which the tight junction protein CLDN6 regulates autophagy. Various proteins participate in autophagy modulation. Beclin1, the first identified mammalian autophagy gene, is a haploinsufficient tumor suppressor and plays an indispensable role in the initiation of autophagy [56–58]. Strikingly, we found that the expression of beclin1 was consistent with that of CLDN6 in breast cancer cells. We also found that the expression of CLDN6 was positively correlated with beclin1 expression in breast cancer tissues. Moreover, silencing beclin1 reduced autophagy and reversed the inhibitory effect of ERβ/CLDN6 on the migration and invasion of breast cancer cells and attenuated the inhibitory effect of CLDN6 on metastasis in vivo. Our results are in line with previous evidence that autophagy inhibition upon beclin1 knockdown stimulates the migration and invasion of GL15 glioma cells [59]. Deletion of beclin1 has been shown to promote the invasion and metastasis of breast cancer cells by increasing the phosphorylation of AKT and ERK [60]. Furthermore, in this study, we found that the expression of ZO-1 and UVRAG was increased in CLDN6-overexpressing cells. Co-IP assays revealed that ZO-1 and UVRAG had binding affinities for CLDN6 and beclin1. Our results demonstrated that the scaffold protein ZO-1 and the autophagy regulatory protein UVRAG functioned as bridge molecules for the CLDN6-beclin1 interaction. This is the first study to report that CLDN6 and ZO-1/UVRAG/beclin1 form complexes with other autophagy proteins to regulate autophagosome formation in breast cancer.

## Conclusion

**Fig. 8:**
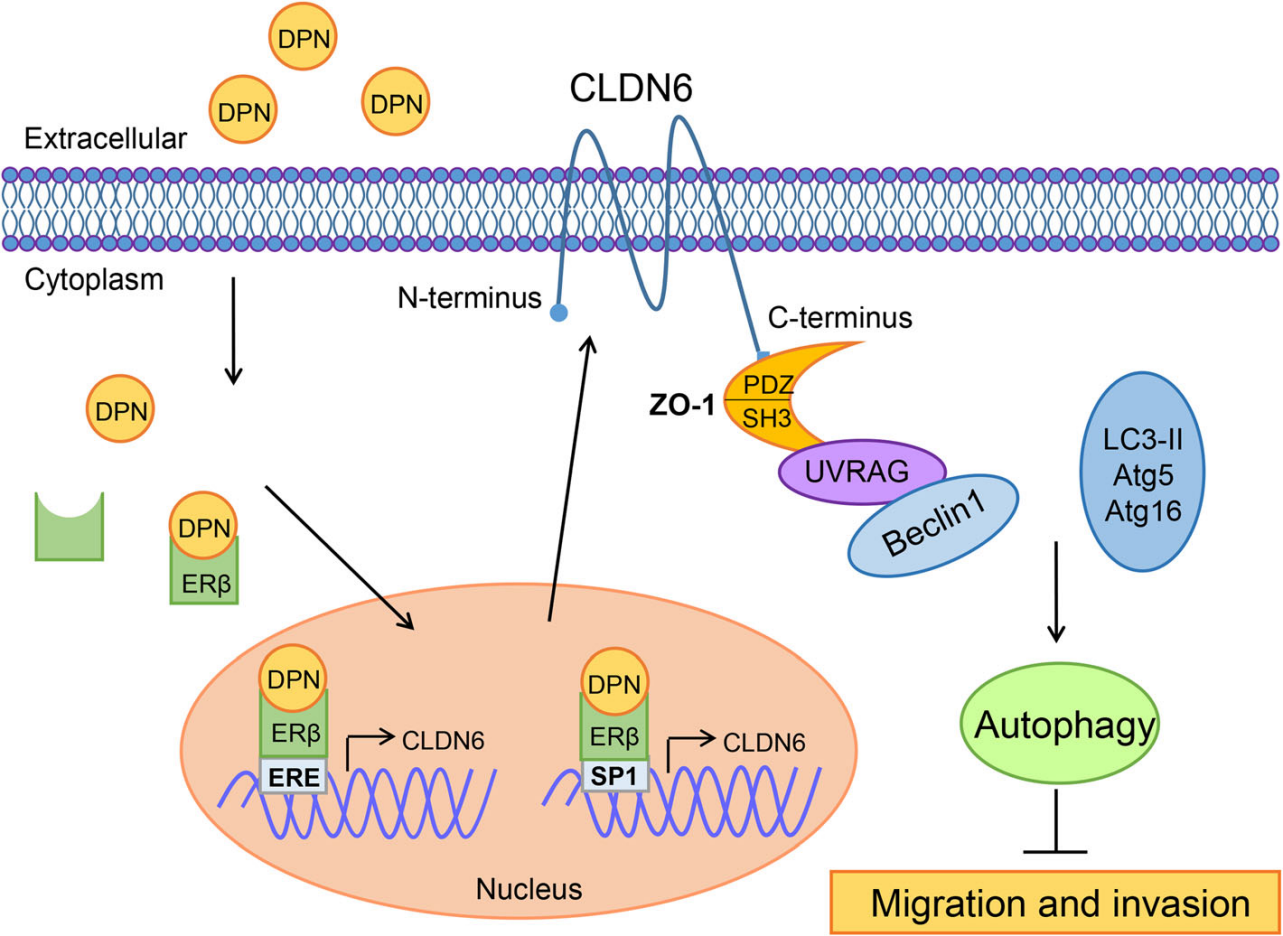
The proposed model for ERβ-induced autophagy inhibiting breast cancer cell migration and invasion. In this model, when ERβ bound with ligands (DPN), DPN-ERβ complexes directly bound to the ERE of the CLDN6 promoter and enhanced CLDN6 expression. In addition, activated ERβ can interact with Sp1 and bind the Sp1 transcriptional regulation domains of the CLDN6 promoter to induce CLDN6 expression. ZO-1 and UVRAG act as bridge molecules for the CLDN6-beclin1 interaction. CLDN6 and ZO-1/UVRAG/beclin1 form complexes and serve as a platform for recruiting other autophagy regulatory proteins (atg5, atg16 and LC3-II) and induce autophagy to suppress the migration and invasion of breast cancer cells

Our study reported new findings that CLDN6 is a target gene of ERβ. Mechanistically, we demonstrated that the inhibitory effects of ERβ on the migration and invasion of breast cancer cells were mediated by CLDN6, which induced the beclin1-dependent autophagic cascade (Fig. 8). These findings provide fresh insight into the mechanism underlying the inhibitory effects of ERβ on breast cancer. Moreover, high ERβ, CLDN6 or beclin1 expression predicted a favorable prognosis in breast cancer patients. ERβ agonists and CLDN6 may be novel therapeutic approaches for the treatment of breast cancer. More in vivo experiments are needed to validate these findings.

## Additional files

Additional file 1: **Figure S1**. Western blot analysis of CLDN6 and ERβ expression after transfection with the indicated plasmids. (A) CLDN6 knockdown efficiency was detected in DPN-treated MDA-MB-231 cells by western blot. (B) Western blot analysis of ERβ expression after transfection with ERβ cDNA in SK-BR-3 and MDA-MB-231 cells. Data are presented as mean ± SD. The data shown are representative results of three independent experiments. *P < 0.05, **P < 0.01. (TIF 305 kb)

Additional file 2: **Figure S2**. DPN regulates CLDN6 expression through ERβ. (A) LC3B protein expression was detected by western blot in DPN-treated MDA-MB-231 cells infected with ERβ-shRNA. (B) The numbers of LC3II puncta (white arrowheads) were decreased after knocking down ERβ in DPN-treated MDA-MB-231 cells as observed in immunofluorescence analysis. Nuclei were stained with DAPI (Scale bar, 20 μm). Data are presented as mean ± SD. The data shown are representative results of three independent experiments. **P < 0.01, ***P < 0.001. (TIF 2134 kb)

Additional file 3: **Figure S3**. Western blot analysis of CLDN6 expression after transfection with the indicated plasmids. (A) Western blot analysis of CLDN6 expression in DPN-treated MDA-MB-231 and SK-BR-3/ERβ cells. CLDN6 knockdown efficiency was detected by western blot in DPN-treated MDA-MB-231 and SK-BR-3/ERβ cells. (B) CLDN6 overexpression efficiency was detected in MDA-MB-231, SK-BR-3 and MCF-7 cells by western blot. Data are presented as mean ± SD. The data shown are representative results of three independent experiments.*P < 0.05, **P < 0.01. (TIF 476 kb)

## Data Availability

The datasets used and/or analyzed during the current study are available from the corresponding author on reasonable request.

## Abbreviations

3-MA: 3-Methyladenine
AKT/PKB: Protein Kinase B
AP1: Activating protein-1
ChIP: Chromatin immunoprecipitation
CQ: Chloroquine
DAB: 3, 3′-diaminobenzidine
DAPI: 4, 6-diamino-2-phenylindole
DFS: Disease Free Survival
DPN: Diarylpropionitrile
E2: 17β-estradiol
ERE: estrogen response element
ERK: Extracellular signal-regulated kinase
GPER1/GPR30: G protein coupled estrogen receptor 1
H&E: Hematoxylin and Eosin
ICI: ICI 182.780
IHC: Immunohistochemistry
OS: Overall Survival
PBS: Phosphate Buffer Saline
shRNAs: Short hairpin RNAs
Sp1: Stimulating protein 1
TEM: Transmission Electron Microscopy
TJs: Tight Junctions
UVRAG: UV-radiation Resistance Associated Gene
ZO-1: Zonula Occludens-1
