# Supplementary figures and images for: Estrogen receptor β inhibits breast cancer cells migration and invasion through CLDN6-mediated autophagy
Source: J Exp Clin Cancer Res. 2019 Aug 14;38:354. doi: 10.1186/s13046-019-1359-9 (PMC6694553; doi:10.1186/s13046-019-1359-9)

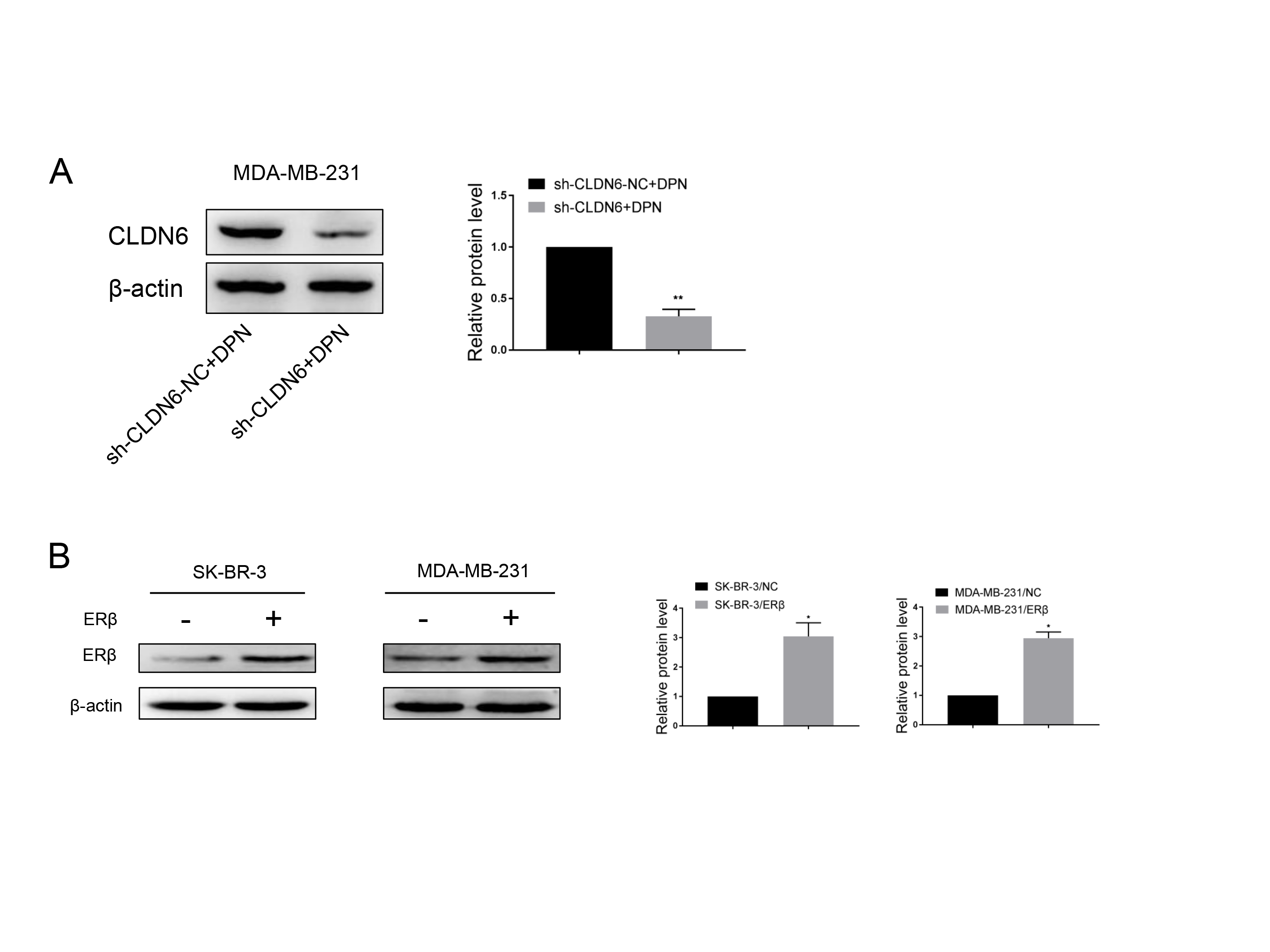

Supplement: Supplementary file 1 — Figure S1. Western blot analysis of CLDN6 and ERβ expression after transfection with the indicated plasmids. (A) CLDN6 knockdown efficiency was detected in DPN-treated MDA-MB-231 cells by western blot. (B) Western blot analysis of ERβ expression after transfection with ERβ cDNA in SK-BR-3 and MDA-MB-231 cells. Data are presented as mean ± SD. The data shown are representative results of three independent experiments. *P < 0.05, **P < 0.01. (TIF 305 kb) [file 13046_2019_1359_MOESM1_ESM.tif]

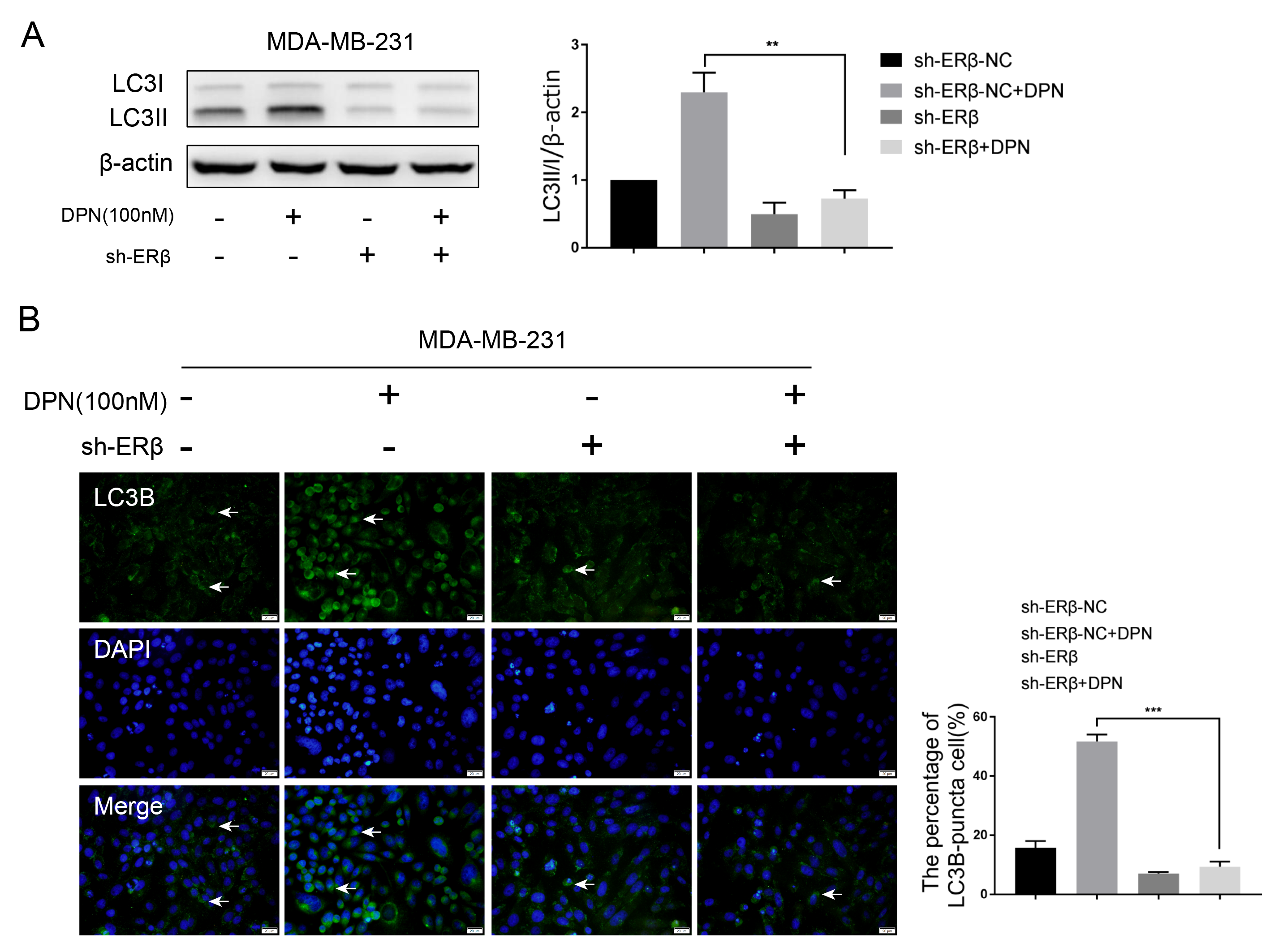

Supplement: Supplementary file 2 — Figure S2. DPN regulates CLDN6 expression through ERβ. (A) LC3B protein expression was detected by western blot in DPN-treated MDA-MB-231 cells infected with ERβ-shRNA. (B) The numbers of LC3II puncta (white arrowheads) were decreased after knocking down ERβ in DPN-treated MDA-MB-231 cells as observed in immunofluorescence analysis. Nuclei were stained with DAPI (Scale bar, 20 μm). Data are presented as mean ± SD. The data shown are representative results of three independent experiments. **P < 0.01, ***P < 0.001. (TIF 2134 kb) [file 13046_2019_1359_MOESM2_ESM.tif]

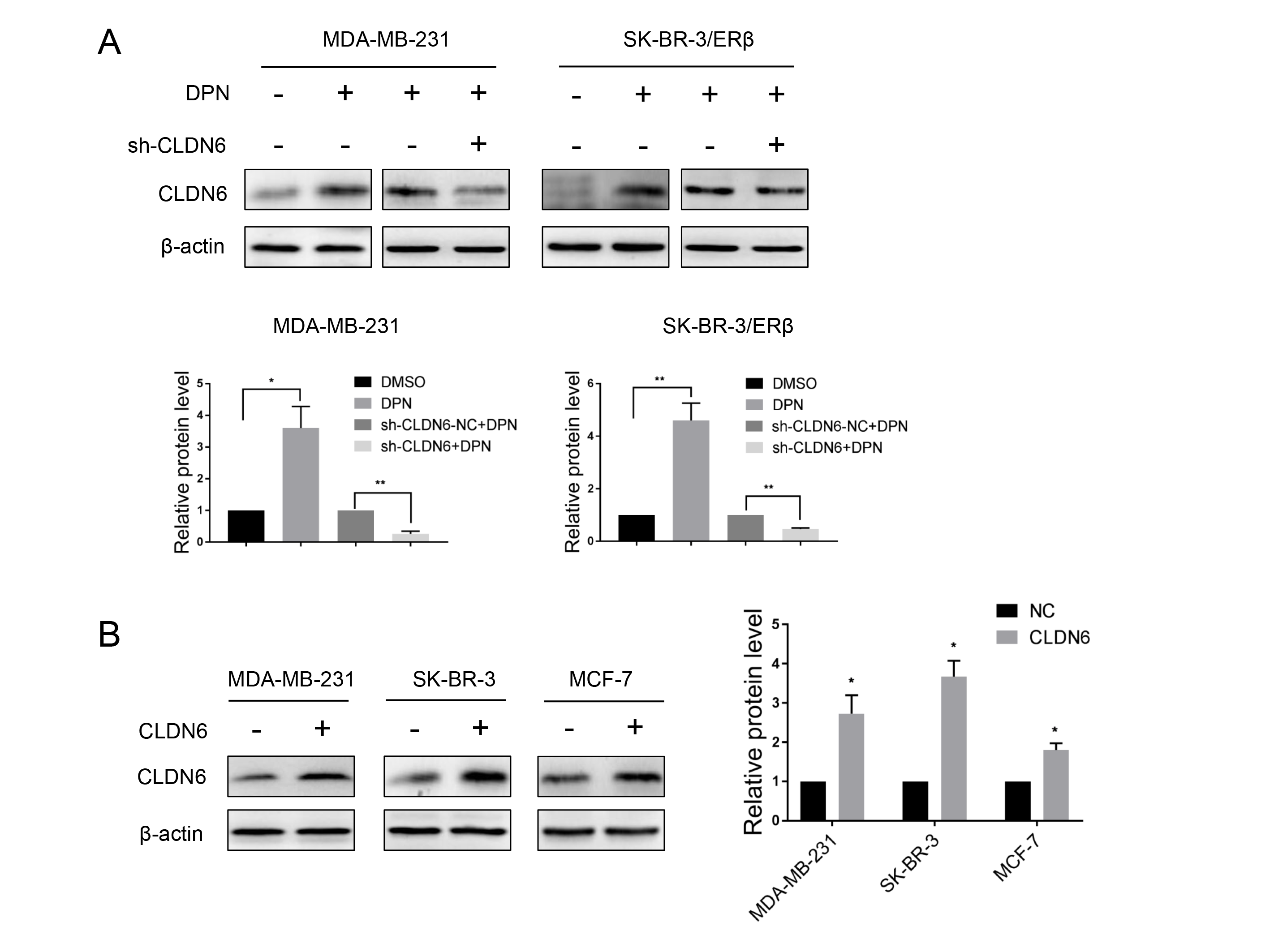

Supplement: Supplementary file 3 — Figure S3. Western blot analysis of CLDN6 expression after transfection with the indicated plasmids. (A) Western blot analysis of CLDN6 expression in DPN-treated MDA-MB-231 and SK-BR-3/ERβ cells. CLDN6 knockdown efficiency was detected by western blot in DPN-treated MDA-MB-231 and SK-BR-3/ERβ cells. (B) CLDN6 overexpression efficiency was detected in MDA-MB-231, SK-BR-3 and MCF-7 cells by western blot. Data are presented as mean ± SD. The data shown are representative results of three independent experiments.*P < 0.05, **P < 0.01. (TIF 476 kb) [file 13046_2019_1359_MOESM3_ESM.tif]
